# Debunking the Myth: Eggs and Heart Disease

**DOI:** 10.7759/cureus.59952

**Published:** 2024-05-09

**Authors:** Raymond Haward, Joshua Chacko, Sonal Konjeti, Gurukiran R Metri, Bezalel K Binoy, Rachel Haward, Sony Raju

**Affiliations:** 1 Internal Medicine, Vydehi Institute of Medical Sciences and Research Centre, Bangalore, IND; 2 Internal Medicine, Father Muller Medical College, Mangalore, IND; 3 General Practice, Jawaharlal Nehru Medical College, Hyderabad, IND; 4 Internal Medicine, Bangalore Medical College and Research Institute (BMCRI), Bangalore, IND; 5 Internal Medicine, KVG Medical College & Hospital, Sullia, IND; 6 Emergency Medicine, Holy Family Hospital, Thodupuzha, IND

**Keywords:** propaganda, healthy life, ldl cholesterol, hdl-cholesterol, hdl ldl, st-elevation myocardial infarction (stemi), heart disease, egg albumin, egg white, fried egg

## Abstract

Eggs, which are often considered a complete food, have recently been scrutinized by the media as a potential cause of cardiovascular disease. However, the media hasn't shown the same enthusiasm for processed foods high in fructose, the consumption of refined cooking oil, seed oils, and carbohydrate-rich meals, the connection between these factors and metabolic diseases, or the potential long-term impacts on population comorbidities, as they have for criticizing egg yolks as a cause for cardiovascular disease in recent times. This review investigates the relationship between eggs and lipid levels, glucose levels, atherosclerosis, and antioxidant properties, as well as comparing them to cholesterol-free egg controls.

We conducted the review in response to a recent trend of discarding nutritious and energy-rich egg yolks due to the belief propagated by the media that removing egg yolks from a normal diet is cardioprotective after the media started to blame egg yolks as the cause of the recent surge in heart attacks. However, the media fails to highlight the fact that eggs have been an integral part of the human diet since the domestication of hens. On the other hand, recent additions to the human diet a few decades ago, such as fructose-rich breakfast cereals, coffee beverages with sugar levels comparable to candy bars, protein supplements for diabetics that are notorious for raising blood glucose levels, and the heightened consumption of seed oil, which causes inflammation, have been responsible for the surge in cardiovascular events in recent times. Social media platforms often showcase visually appealing junk food products and sugary beverages as a sign of wealth, promoting unhealthy processed food and ultimately causing a decline in an individual's lifespan and overall health.

## Introduction and background

Atherosclerotic cardiovascular disease (CVD) remains the leading cause of mortality in the world and is responsible for a staggering number of deaths annually, with approximately 19 million fatalities attributable to heart disease each year [1]. Healthcare workers find it increasingly challenging to care for patients with atherosclerotic cardiovascular disease (CVD), particularly in light of the processed food industry's promotion of unhealthy, yet tastier, processed foods sold at a subsidized cost. Several studies over the years have shown that lowering risk factors like smoking, high blood pressure, diabetes, hypothyroidism, and dyslipidemia, along with following a healthy low-carbohydrate diet and doing moderate exercise, can help lower the number of atherosclerotic CVD events [2]. The environment we live in often makes it difficult for patients to sustain their treatment goals over time. Advertisements, such as big discounts at a new fast-food restaurant, the culture of frequent parties and gatherings with deep-fried and carbohydrate-rich food, and the belief in occasional cheat meals, promoted by social media, all contribute to this.

**Figure 1:**
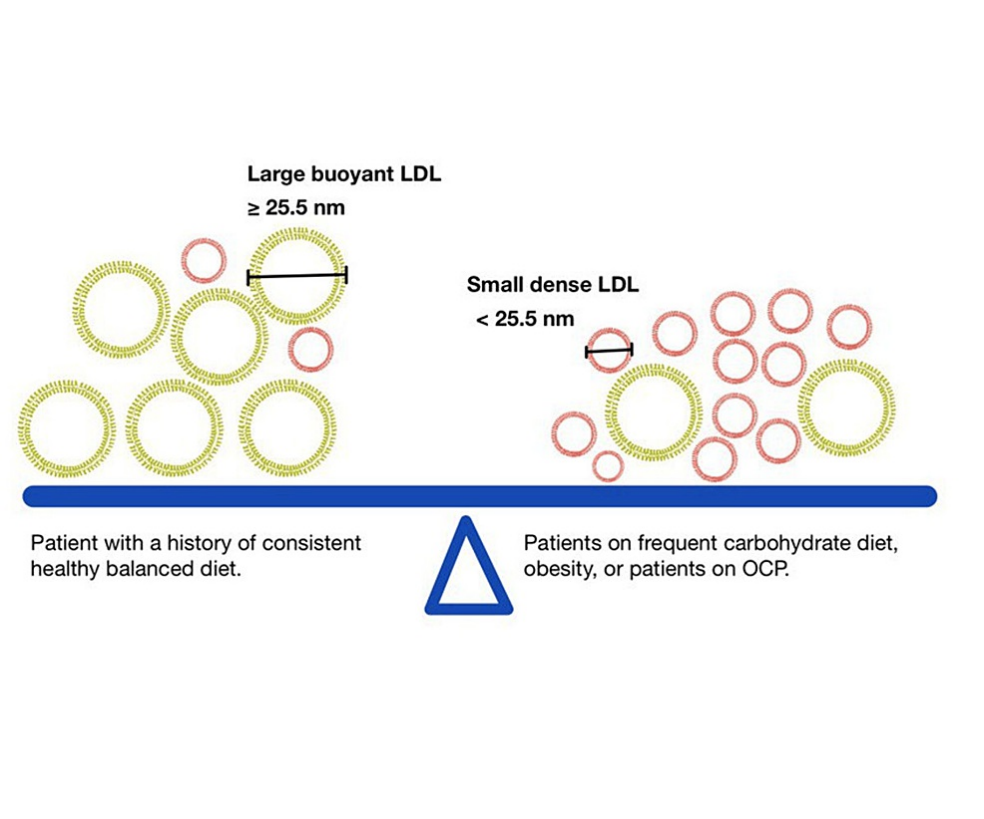
Visualizing the Distribution of Large Buoyant LDL and Small Dense LDL in Patients with Healthy and Unhealthy Diet This figure has been created by the authors. LDL: Low-density lipoprotein; OCP: Oral contraceptive pill

The pursuit of lowering low-density lipoprotein (LDL) cholesterol levels below specified thresholds represents a cornerstone in mitigating atherosclerotic CVD risk. Despite the correlation between decreased LDL cholesterol levels and reduced atherosclerotic CVD event rates, a substantial proportion of patients fail to attain these goals even with maximal doses of statins and other hypolipidemia medications [2]. This is due to the fundamentals of the two subtypes of LDL: the large buoyant form and the small dense form [3, 4]. Saturated-fat diets produce the large buoyant form of LDL, controllable by statins and not linked to cardiovascular events [3-5]. Individuals with abdominal obesity, those who frequently consume carbohydrate-rich diets, and those on oral contraceptive pill (OCP) exhibit elevated levels of the smaller dense LDL subtype in their blood, which statins fail to effectively reduce and also increase the cardiovascular risk due to atherosclerosis (Figure 1).

In recent years, the media has speculated that eggs, considered a complete food for centuries, could cause myocardial infarction [6]. There were suggestions for eliminating the egg yolk and eating only egg white as a solution to prevent cardiovascular disease [6]. However, several studies on pooling showed inconclusive results on heart problems [7]. Studies prove that egg-free substitutes have not reduced the risk of CVD when compared to whole eggs [8].

Therefore, we are conducting this review to investigate whether the media's claims about egg consumption and cardiovascular health hold true or not. Furthermore, this study investigates how patients with metabolic disorders improve while eating a predominantly egg diet with carbohydrate restriction [8].

## Review

Unlocking the nutritional value of eggs

Both the egg yolk and the egg white make up a complete egg, with the egg yolk accounting for approximately 33%. Within this portion, 50% is water, while the remainder consists of beneficial lipids, proteins, vitamins, and minerals [9]. A large hard-boiled egg provides around 78 calories, 6.29 grams of protein, 5.3 grams of fat, and 0.56 grams of carbohydrate, contributing to the creamy yellow to orange hue of egg yolks [10].

The lipids in egg yolks encompass a blend of healthy unsaturated fatty acids, including triglycerides, phospholipids, cholesterol, high-density lipoprotein (HDL), low-density lipoprotein (LDL), and very low-density lipoprotein (VLDL) [11]. LDL constitutes two-thirds of the total egg yolk and is responsible for its emulsifying properties, while proteins provide essential amino acids [11].

Despite their small size, egg yolks serve as a significant source of essential vitamins such as A, B2, B6, B12, biotin, folic acid, D, E, and K, along with minerals like calcium, zinc, iron, potassium, magnesium, iodine, fluoride, selenium, and phosphorus [12, 13]. These vitamins and minerals play crucial roles in maintaining vision, contributing to bone and muscle growth, promoting well-being, and strengthening immunity [12, 13]. Additionally, they contain antioxidants such as lutein and zeaxanthin, which support eye health, and phospholipids known for their anti-inflammatory properties [14].

Ratliff et al. conducted a three-month study on patients with metabolic syndrome and found that consuming three eggs per day reduced tumor necrosis factor and C-reactive protein (CRP) levels compared to the placebo group, demonstrating the anti-inflammatory effects of eggs [15].

Phosvitin is an egg-derived peptide, and amino acids such as tryptophan and tyrosine found in egg yolk have potent antioxidant properties [16]. Furthermore, studies have demonstrated the protective characteristics of phosvitin against DNA damage, as well as its cytotoxic properties against human cancer cell lines like AGS (stomach), MCF-7 (breast), HeLa (cervix), and HepG2 (liver) [16]. Furthermore, livetin and IgY, found in egg yolk, exhibit immunomodulatory and anti-cancer activities [16]. IgY binds tightly to the DR5 protein, which activates the apoptotic effect of the TNF-related apoptosis-inducing ligand in cancer cells [17]. This leads to the apoptosis of MCF-7 cells. Therefore, the presence of antioxidants, vitamins, minerals, and immune-modulatory properties in nutrient-dense eggs makes them a valuable addition to a balanced diet. Moreover, incorporating eggs into various dishes enhances their versatility and offers beneficial food options for overall health.

Exploring the connection: eggs and cardiovascular well-being

Researchers widely recognize high levels of LDL as a risk factor for cardiovascular disease. The researchers at the Framingham Heart Study were the first to establish the correlation between high cholesterol levels and cardiovascular disease (CVD) risks. As a result, they determined that maintaining healthy levels of blood cholesterol is crucial for a healthy heart [18]. Nevertheless, this study [18] did not discover any correlation between the consumption of food containing cholesterol and increased levels of cholesterol. Furthermore, a rise in total cholesterol levels may not accurately show the risk of atherosclerosis because the cholesterol profile also includes high-density lipoprotein (HDL), which protects against atherosclerosis [8]. The Framingham study has not shown any connections between blood cholesterol and several health outcomes, including all-cause mortality, coronary artery disease, and myocardial infarction [18]. Hozawa et al. provided estimates on the impact of increasing dietary cholesterol on LDL, HDL, and total cholesterol levels. Findings indicated that for every 100 mg increase in dietary cholesterol, there is a corresponding increase in blood levels of total cholesterol by 2.2 to 2.5 mg/dl, LDL by 1.9 mg/dl, and HDL by 0.4 mg/dl [19]. Subsequent studies have demonstrated a negative correlation between the intake of cholesterol and the composition of lipids in the bloodstream. Individual differences in the body's processing of dietary fats within the population may account for this relationship [7, 20].

Following the consumption of dietary cholesterol, two distinct responses have been identified: normal responders/hypo responders and hyper responders [21]. Approximately 75% of the population exhibit moderate to minimal changes in their plasma cholesterol levels when consuming dietary cholesterol and are categorized as normal responders or hypo responders [21]. This phenomenon may be caused by the gut's absorption of only a fraction of the dietary cholesterol, or a reduction in endogenous cholesterol synthesis due to increased dietary cholesterol intake [22]. Notably, minimal alterations occur in individuals consuming a cholesterol-rich diet, as the ratios of LDL and HDL remain balanced with the increase in dietary cholesterol, proportionally elevating HDL levels. Conversely, individuals experiencing elevated HDL and LDL levels following increased dietary cholesterol intake are classified as hyper responders [21].

Meta-analyses conducted on prospective cohort studies have not conclusively established a direct link between coronary artery disease (CAD) and egg yolk consumption. Recent observations suggest that the statistical significance of dietary data can change depending on the statistical methods used for analysis [19]. This can change the results of the studies that use those methods. We urge researchers to carefully interpret results and select unbiased statistical methodologies to accurately assess diet-disease relationships [19].

Mutungi et al. observed a significant increase in HDL levels in individuals who consume eggs alongside moderate carbohydrate restriction [23]. There is potential for further investigation into the effects of egg consumption among individuals with normal carbohydrate intake, particularly since low carbohydrate diets have been associated with reduced triglyceride levels in the bloodstream [8]. Potent antioxidants like lutein and zeaxanthin, known to protect against lipid oxidation and prevent atherosclerosis, may account for the apparent absence of evidence suggesting harm from egg yolk consumption. Additionally, the concept of "normal responders," constituting approximately 75% of the population, who are capable of maintaining steady lipid levels, warrants further exploration [24].

Geographical locations, ethnicities, and dietary practices, including veganism, influence variations in egg consumption. This led to an examination of egg consumption within the Mexican population, where eggs play a significant role in their diet [25]. Notably, Mexicans have a high prevalence of dyslipidemia and are at increased risk of cardiovascular events [25]. Research involving Mexican children consuming two eggs per day for 30 days revealed a higher proportion of large, less atherogenic LDL particles compared to those consuming a cholesterol-free egg substitute. The widespread availability and affordability of carbonated soft drinks, with Mexico ranking as the second-largest global consumer, may exacerbate the prevalence of dyslipidemia in the country [25, 26].

Researchers conducted similar investigations with different demographic groups. For instance, a study involving college students consuming two eggs five times a week for 14 weeks showed no change in the LDL/HDL ratio [27]. Likewise, studies on postmenopausal women and adults aged 40 to 65 who consumed one or three eggs daily demonstrated no significant impact on lipid profiles, challenging the notion that egg yolks are inherently unhealthy [28, 29]. In fact, evidence suggests that egg consumption may even offer benefits, particularly when combined with regular exercise. Patients engaging in exercise three times a week while consuming two eggs daily for 12 weeks experienced decreased triglyceride levels compared to controls [30].

The concern over egg yolks contributing to atherosclerosis was hypothesised due to their rich choline content. Studies on both animals and humans have linked dietary choline to cardiovascular diseases. This is because bacteria in the gut turn choline into trimethylamine N-oxide (TMAO) [31]. Researchers further explored this finding by measuring serum TMAO levels following consumption of whole eggs, egg whites, or choline bitartrate supplements. Findings stated that eating two to three eggs a day did not significantly change baseline TMAO levels, but taking supplements increased TMAO levels [32-34]. Similarly, studies on overweight postmenopausal women consuming two eggs daily for four weeks showed no effect on TMAO concentrations, suggesting that egg-based choline does not promote atherosclerosis [35].

Research conducted on obese individuals following a low-fat, energy-deficit diet (1000 kcal) revealed that a breakfast comprising two eggs exhibited no discernible variance in serum lipid levels compared to a bagel-based breakfast [36]. However, a significant decrease in adiposity was observed in those consuming the egg-based breakfast in contrast to the bagel-based option, indicating that substituting eggs in breakfast alongside regular dietary intake contributes to adiposity reduction. In a similar vein, a 12-week study incorporating three eggs per day within a moderately carbohydrate-restricted diet demonstrated an elevation in HDL levels without affecting plasma cholesterol [23]. Conversely, a separate investigation involving the consumption of four eggs daily showed a proportional increase in both HDL and LDL [37]. Consequently, current research fails to implicate eggs as the sole instigator of heart diseases. Additionally, research involving people with type 2 diabetes mellitus showed that eating one egg every day for five weeks lowered levels of inflammatory mediators like TNF-alpha and CRP when compared to a breakfast of oatmeal [38].

HDL serves as a biomarker for assessing cardiovascular disease (CVD) [39]. Lower levels of HDL have been associated with an increased risk of cardiovascular disease [39]. On the other hand, having more HDL particles makes it easier for macrophage foam cells to remove lipids, which reverses atherosclerosis in heart vessels [39].

Researchers Andersen et al. found that feeding RAW 264.7 macrophage cells from people with metabolic syndrome three eggs a day for 12 weeks increased their capacity to accept cholesterol by week 12 compared to controls fed cholesterol-free foods [40]. This is due to an increased level of an enzyme called lecithin cholesterol acyltransferase levels, which improved the cholesterol-accepting capacity [40]. This study also found that eating whole eggs with lipopolysaccharide protected against inflammatory cytokines in mononuclear cells better than serum from cholesterol-free egg substitutes [40].

Antioxidants like lutein and zeaxanthin significantly elevate in serum levels after egg ingestion, providing protection against inflammation, oxidation, and atherosclerosis [41]. Although the precise mechanisms underlying the cardioprotective effects of eggs remain unclear, it is generally considered safe to consume eggs without increasing the risk of CVD in both healthy individuals and those with existing conditions [41].

Studies examining the relationship between egg intake and glucose metabolism have yielded mixed results. In experiments conducted on Wistar albino rats, feeding on egg yolk cholesterol led to hyperglycemia compared to a control diet [42]. Similarly, studies by Zutphen and Djousse demonstrated a positive correlation between egg consumption and fasting glucose levels [43, 44]. However, a prospective study involving 4000 participants over the period of 1989-2007 found no association between egg consumption and type 2 diabetes [44]. Additionally, Pearce et al.'s research observed no impaired glucose mechanisms when participants consumed three eggs daily under carbohydrate restriction for 12 weeks [45]. The effects of egg consumption on glucose metabolism in diets predominantly consisting of carbohydrates and those with restricted carbohydrate intake warrant further investigations involving both humans and animals.

Studies have also explored the relationship between stroke risk and egg consumption, given that strokes rank as the fourth most common cause of heart disease and frequently occur in patients with cardiovascular diseases. Research by Scrafford et al., analyzing data from the National Health and Nutrition Examination Survey (NHANES III), found no association between consuming six eggs per week and stroke [46]. Similarly, a prospective meta-analysis by Rong et al. concluded that consuming one egg per day did not increase the risk of stroke [20].

Controversies, colluding and conflicting studies

There is still disagreement about the link between eating egg yolks and the risk of heart disease because of the complicated interaction of many factors, such as dietary habits, stress levels, how people react to dietary cholesterol (for example, hyperresponders vs. normoresponders), and the study parameters. A contributing factor to this uncertainty could be the dangers associated with mindless emotional eating, a concern so significant that even GlaxoSmithKline, a pharmaceutical giant, intends to produce a documentary highlighting its hazards [47]. Furthermore, inconsistencies may also arise from suspicions of conspiracy, wherein powerful food and pharmaceutical industries, conducting studies, manipulate outcomes to undermine the purported benefits of egg consumption [48].

Because of extensive efforts by medical associations, awareness of the dangers of processed foods has grown significantly. Consequently, the food industry often employs subtle tactics to promote articles on natural foods, highlighting their drawbacks to foster the perception that all foods possess inherent disadvantages and occasional consumption of processed foods is acceptable [49]. Furthermore, the food industry's environment, characterized by enticing advertisements offering extra cheese, heightened spiciness, and discounted prices compared to market rates, further exacerbates the issue [50]. These marketing strategies permeate various platforms, including shopping malls, condiment stores, and the media, ultimately serving not only to bolster profits for the food industry but also to cultivate future clientele for the pharmaceutical industry [51].

Methodological differences, population diversity, and confounding variables complicate the interpretation of study results concerning egg yolk consumption and cardiovascular risk [52]. For instance, a study from Japan found that daily egg intake correlated with a 30% lower risk of overall stroke mortality compared to rare egg consumption [53]. However, limitations, such as a small sample size and insufficient cases among stroke subtypes, undermine the reliability of these findings. Additionally, a meta-analysis by Alexander et al. in 2016 found no significant associations between cardiovascular disease and stroke across seven prospective studies [54]. In contrast, a study by Qin et al. involving 0.5 million Chinese individuals revealed a strong link between cardiovascular disease and egg yolk consumption [55]. Carter et al. highlighted population-specific factors as the reason for the disparity between these findings [56].

Furthermore, assessing the isolated effects of egg yolks on cardiovascular health is complicated by dietary patterns and individual variations in cholesterol metabolism and genetic predispositions [57, 58]. Resolving the controversies surrounding egg yolk consumption is challenging due to the multifactorial nature of cardiovascular risk, complexities in dietary intake, and metabolic responses. Moreover, the presence of refined carbohydrates and seed oils in daily diets hampers the potential health benefits of eggs, with refined oils increasing the omega-6/omega-3 ratio and promoting inflammation [59].

In recent decades, the concept of "cheat days" has emerged, promoting unhealthy, fructose-rich processed foods that can disrupt a consistent healthy diet [60]. These foods contribute to increased uric acid levels and inhibit nitric oxide production, affecting vascular health [61]. Moreover, people often overlook fructose's role in promoting non-alcoholic fatty liver disease (NAFLD) [62].

The marketing strategies of food industries exacerbate these issues, promoting protein powders and bars that may worsen blood sugar control and contribute to obesity [63-65]. Similarly, coffee beverages loaded with sugar mimic candy bars, contributing to sugar addiction and obesity [66, 67]. Breakfast cereals marketed to children often contain high levels of fructose and refined oils, contributing to future health problems [68]. The promotion of processed foods as "smart choices" perpetuates misconceptions about their healthfulness [69].

Despite these challenges, the nutritional value of eggs has received limited promotion, overshadowing their benefits [70]. Reinforcing the health benefits of eggs is essential to promote a balanced diet and combat obesity, given their high satiety and overall nutritional value.

Future directions

Future research should prioritize investigating cholesterol absorption capacity, particularly the influence of ABCG5 gene polymorphism. Notably, the C/C genotype is linked to heightened levels of LDL and cholesterol in plasma compared to C/G and G/G genotypes [71]. Expanding on this, there is a pressing need for studies to elucidate the metabolic processes involved in fat digestion and the body's tolerance to fatty foods under varying conditions. Additionally, anti-inflammatory compounds like phosvitin, lutein, and zeaxanthin must be further explored to determine their long-term efficacy.

Further investigation is necessary to determine the optimal quantity of egg consumption per session for improved health outcomes, taking into account factors such as a regular versus a carbohydrate-deficient diet. Delving into the relationship between egg yolk and glucose metabolism is essential to determine whether the predominant response to egg consumption among patients with type 2 diabetes mellitus is protective or inconsequential.

Finally, large-scale research projects should examine a variety of situations, such as differences in the number of eggs eaten, differences in how people react to eating eggs when they follow different eating patterns, and how dietary lipids affect egg intake. Addressing these aspects, especially in patients with known comorbidities, is crucial to harnessing the health benefits derived from nature for a robust cardiovascular system.

## Conclusions

Eggs are incredibly healthy and make a great contribution to a balanced diet. They have antioxidants and notably decrease triglyceride levels. Egg-predominantly carbohydrate-deficient meals show the best improvement in lipid levels when compared to similar eggless controls. Avoid discarding egg yolks, as they contain antioxidants, essential fatty acids, proteins, vitamins, and minerals that contribute to a healthy and balanced diet. Also, consumers must be aware of the media game, which portrays the illusion that eating their highly processed junk food is not as bad because the healthy egg yolks are also bad, despite the truth being that egg yolks are good for health.
